# Effects of Pretreated Phosphogypsum and Granulated Blast-Furnace Slag on the Rheological Properties of the Paste Excited by NaOH

**DOI:** 10.3390/molecules28062662

**Published:** 2023-03-15

**Authors:** Shuangkuai Dong, Songhan Yu, Liangliang Chen, Qi Zhuo, Fufei Wu, Lilan Xie, Liuyan Liu

**Affiliations:** 1 School of Materlals and Architectural Engineering (Guizhou School of Emergency Management), Guizhou Normal University, Guiyang 550025, China; 2 School of Civil Engineering, Huzhou Vocational and Technical College, Huzhou 313000, China; 3 Huzhou Key Laboratory of Green Building Technology, Huzhou 313000, China

**Keywords:** phosphogypsum, granulated blast-furnace slag, rheological property, membrane thickness, optimize

## Abstract

The main component of phosphogypsum (PG) is CaSO_4_·2H_2_O. PG contains a few impurities, heavy metals, and radioisotopes, which limit the use of PG and pose a danger to the environment. In this study, under the excitation of a sodium hydroxide solution, the rheological properties of a paste with granulated blast-furnace slag (GGBS) and PG treated with ultrasonic water washing were investigated. Experimental results showed that the ratio of GGBS to PG and the amount of sodium hydroxide solution significantly affect the density and viscosity of the paste, but the effect patterns of both are different. The maximum viscosity was 498 mPa·s when the ratio of GGBS to PG was 4:1. When the ratio changed from 3:2 to 1:4, the viscosity of the paste gradually decreased by 15.5%, 32.1%, 36.1%, and 46.8%, respectively. In contrast, the ratio of GGBS to PG had a greater effect on the viscosity than the amount of sodium hydroxide solution in terms of the standard consistency water consumption, viscosity, and water release ratio. The larger the PG ratio, the smaller the density, viscosity, and water release ratio of the paste. The variation in the ratio of GGBS to PG had a significant effect on the water film thickness of the paste, demonstrating that the larger the PG mixture, the larger the water film thickness of the paste, which reached 1.122 μm, 2.31 times the minimum water film thickness of the paste. At the same time, the water film thickness of the paste was negatively correlated with the water consumption of the standard consistency, viscosity, and water release ratio, and was positively correlated with the fluidity.

## 1. Introduction

Cement-based materials are the most widely used and most abundant construction materials in the world [1,2]. However, the production of cement-based materials causes serious environmental pollution and destruction [3,4], especially in the production process of cement clinker, which is more serious. First, the production of cement clinker must consume a significant amount of limestone and clay, and their exploitation has seriously damaged the natural environment. Second, the “two mills and one burn” process of producing cement clinker requires the consumption of a large amount of energy. Finally, the decomposition of limestone and the consumption of electricity and coal in the cement production process all lead to increased carbon emissions [5]. Therefore, an increasing number of experts and scholars are calling for the implementation of carbon tax policies to promote the transformation of the cement industry and reduce carbon emissions. It is noteworthy that after proper pretreatment, many industrial solid wastes in Guizhou, China, demonstrate a certain pozzolanic activity and even hydraulicity, and can be used as supplementary cementitious materials for cement and concrete [6,7]. In particular, since the German scholar Kuhl proposed alkali-excited cementitious materials, which have the advantages of a low cost, corrosion resistance, high-temperature resistance, and low energy consumption, they have extensively attracted the attention of scholars [8,9]. This can not only provide conditions for their development in the solid waste industry but can also significantly reduce the energy consumption and carbon emissions in the process of producing cement, thus achieving the strategic goal of low-carbon environmental protection.

Phosphogypsum (PG) is an industrial waste produced during the production of wet-process phosphoric acid. Its main component is CaSO_4_·2H_2_O, and it contains organic matters, phosphides, fluorides, heavy metals, and radioactive elements [10,11]. At present, the amount of PG accumulation in China has exceeded 500 million tons and continues to increase at a rate of approximately 50 million tons per year. However, its comprehensive utilization rate is less than 40%, and most of it is mainly stored in pile, which not only wastes land resources [12,13] but also allows the harmful impurities in PG to penetrate the groundwater or flow into rivers and lakes, causing serious water pollution and threatening biological and human health. Therefore, the efficient consumption of PG is of great significance to promote the green and high-quality development of the phosphorus chemical industry. At present, PG is often used to prepare gypsum boards, gypsum bricks, gypsum blocks, and other wall materials. It has some characteristics, such as being lightweight, high-strength, and fireproof and providing thermal insulation and sound insulation, which make it popular [14,15,16]. In addition, because its chemical composition is similar to that of natural gypsum, PG can replace some natural gypsum to produce cement retarder [17]. In recent years, scholars have found that PG contains a great deal of minerals such as SO_3_, CaO, SiO_2_, etc., which are similar to the chemical composition of cement and can replace part of cement as a cementitious material [18]. It is important to improve the working performance of PG-based cementitious materials, which can be compounded by a certain amount of admixtures, such as granulated blast-furnace slag [19], fly ash [20], silica fume [21], steel slag [22], and other common admixtures, to achieve a comprehensive use of PG. However, impurities such as soluble phosphorus, eutectic phosphorus, and soluble fluorine in PG limit its wide use; therefore, the pretreatment of PG is particularly important. At present, the commonly used pretreatment methods mainly include water washing, lime neutralization, calcination, ball milling, etc. [23,24]. In addition, the evaluation of radioactive elements (^238^U, ^226^Ra, ^228^Ra, ^210^Pb, ^232^Th, and ^40^K, etc.) in PG and the solidification of heavy metals (Zn, Pb, Cr, Ba, Ni, etc.) has caused difficulty in its reuse [25].

The problem of the impurities, heavy metals, and radioisotopes in PG not only limits the use of PG but also poses a danger to the environment. Therefore, in this study, PG was first subjected to ultrasonic water washing to disperse it evenly in water. It was then dried in an oven at 105 °C for its use in later tests after precipitation. The impurity content of the PG obtained by this pretreatment method was reduced, and the resulting water washing solution can be reused. This not only effectively improves the performance of PG composites but also avoids secondary pollution and the high-cost problem caused by pretreatment. Considering that the PG obtained through this innovative method was not treated with high-temperature autoclaving, its activity is low. Therefore, the pretreated PG was compounded with granulated blast-furnace slag (GGBS) [26,27], and the rheological properties of the composite paste were investigated under the excitation of a NaOH solution. This can provide an experimental reference for the development of alkali-excited, PG cementitious materials.

## 2. Results and Discussion

### 2.1. Density of Composite Material

Density is one of the properties of substances; each substance has a certain density, and its value directly affects the quality, performance, and use of the material.

**Figure 1:**
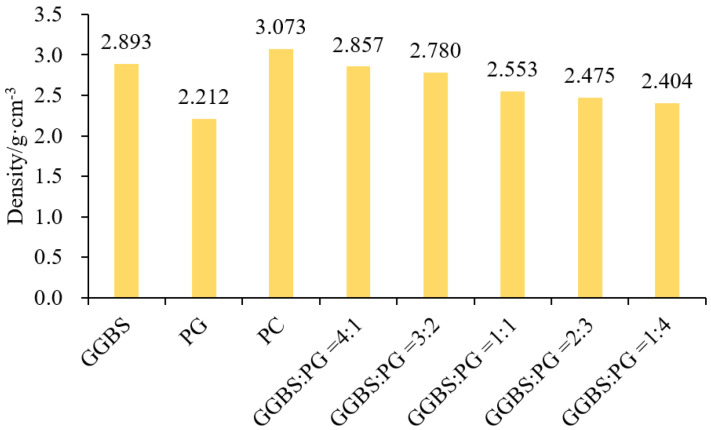
Density of composite material.

The density of different proportions of GGBS and PG composites was tested in the experiment, as shown in Figure 1. It can be seen that the density of the GGBS and PG composites fell between GGBS (2.893 g/cm^3^) and PG (2.212 g/cm^3^), and their densities are related to the ratio of GGBS and PG. Specifically, when the ratio of GGBS to PG was 4:1, the density of the composite powder was the maximum of 2.857 g/cm^3^ and then gradually decreased. Compared with the ratio of 4:1, the density of the composite powder decreased by 2.70%, 10.64%, 13.37%, and 15.86% when the ratio was 3:2, 1:1, 2:3, and 1:4, respectively. That is, with a decrease in GGBS content and an increase in PG content, the density of the composite powder gradually decreases.

### 2.2. Standard Consistency Water Consumption of Composite Material

**Figure 2:**
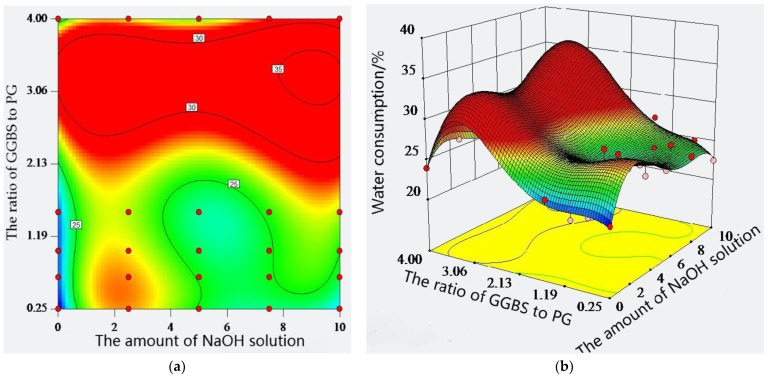
Standard consistency water consumption of composite material: (**a**) contour line; (**b**) three-dimensional surface.

The standard consistency water consumption indicates the thinness of the paste, which is the ratio of water consumption to cement mass when the paste reaches the standard consistency. When too much water is added to the paste, the cement will become thinner and flow easily when smearing. Otherwise, it is not easy to smooth. The standard consistency water consumption of the paste was discussed in the different ratios of GGBS to PG and the amount of sodium hydroxide solution. The results are shown in Figure 2. Comparing cement, GGBS, and PG, it can be found that PG has the smallest water consumption for a standard consistency of 21.5%, followed by cement, with 21.75%. GGBS has the largest consumption at 25.75%. When the amount of sodium hydroxide solution was kept constant, the ratio of GGBS to PG could cause the water consumption of the paste to show ups and downs. For example, at a sodium hydroxide solution dosage of 0%, the standard consistency water consumption of the paste was 24.0%, 23.3%, 21.5%, 22.3%, and 21.8% when the ratio of GGBS to PG was 4:1, 3:2, 1:1, 2:3, and 1:4, respectively. Therefore, when the ratio of GGBS to PG was 4:1, the standard consistency water consumption of the paste reaches a maximum. However, the difference in the standard consistency water consumption was only 2.5% in the different ratios of GGBS to PG. After adding the sodium hydroxide solution at 2.5%, 5%, 7.5%, and 10%, a similar rule still existed, and the difference was no more than 4% with the same dosage of sodium hydroxide solution. Compared with the control group, the difference in the standard consistency water consumption reached a maximum of 6.4% and appeared when the sodium hydroxide solution was 2.5%. Additionally, there is no evidence that the greater the dosage of sodium hydroxide solution, the greater the difference. On the whole, the amount of sodium hydroxide solution also had an ascending trend with respect to the standard consistency water consumption of the paste. However, it was not significant. When the ratio of GGBS to PG was constant, the water consumption increased first, then decreased, and then increased again with an increase in the sodium hydroxide solution. For example, when the ratio of GGBS to PG was 4:1 and the dosage of sodium hydroxide solution was 0%, 2.5%, 5%, 7.5%, and 10%, respectively, and the corresponding water consumption of standard consistency was 24.0%, 26.0%, 25.3%, 27.9%, and 27.2%, similar to other ratios. In addition, from the contour map in Figure 2, it can be seen that there is no significant difference in the density of contour line for the horizontal and vertical coordinate. Therefore, the ratio of GGBS to PG and the amount of sodium hydroxide solution have a similar effect on the standard consistency water consumption of the paste. From the three-dimensional surface, it can be seen that the maximum value of the standard consistency water consumption corresponds to the highest point of the response surface. This indicates that when the amount of the sodium hydroxide solution is 9.76% and the ratio of GGBS to PG is 0.74, the standard consistency water consumption can be optimized to reach the highest value within the test range, and the desirability of the solution is 1.

### 2.3. Viscosity of Composite Material

**Figure 3:**
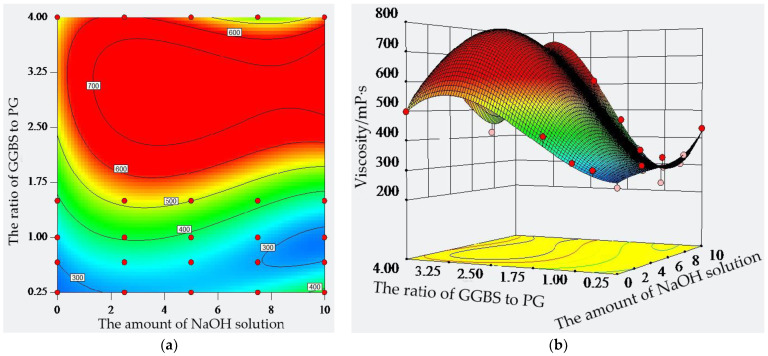
Viscosity of composite material: (**a**) isohypse contour; (**b**) three-dimensional surface.

The viscosity of the paste is the plastic viscosity when flow occurs by shear, and it is an important parameter for studying the rheology of the paste. The composition, structure, and external condition of the paste were simulated to simulate the rheological properties of the paste on the basis of ensuring the accuracy of the viscosity. This experiment studied the effect of the ratio of GGBS to PG and the amount of sodium hydroxide solution on the viscosity of the paste. The results are shown in Figure 3.

When comparing cement, GGBS, and PG, the viscosity of PG is 722 mP·s, which is the largest, and the viscosity of the cement is the smallest at 253 mPa·s. Therefore, it can be seen that the viscosity characteristics of these three materials differ significantly. When the ratio of GGBS to PG was 4:1, the viscosity of the paste reached a maximum value of 498 mPa·s. With the change in the ratio of GGBS to PG from 4:1, 3:2, 1:1, 2:3, to 1:4, the viscosity of the paste gradually decreased by 15.5%, 32.1%, 36.1%, and 46.8%, respectively, compared with the test group, which had a ratio of GGBS to PG of 4:1. It can be found that the viscosity of the paste in the ratio of 4:1 was close to the viscosity of pure GGBS paste, indicating that GGBS plays a leading role in the test paste. When the ratio of GGBS to PG was 1:4, the viscosity of the test paste was not only much lower than the viscosity of pure GGBS paste but was also close to the viscosity of pure PG paste, indicating that the composite effect of GGBS and PG has a certain effect on the viscosity of the paste. When the ratio of GGBS to PG is kept constant, the amount of sodium hydroxide solution can cause the viscosity of the paste to show a downward trend after rising. For example, the viscosity of GGBS to PG in a ratio of 4:1 and an amount of sodium hydroxide solution of 2.5% is the largest. When the GGBS to PG ratios were 3:2, 1:1, and 2:3, and the amount of the sodium hydroxide solution was 5%, the viscosity of the paste was at a maximum of is 604 mPa·s, 470 mPa·s, and 366 mPa·s, respectively. It can be found that the paste viscosity is not only related to the amount of sodium hydroxide solution but also to the ratio of GGBS to PG. When the amount of GGBS is less and the amount of PG is more, the decrease in the enhancement effect of the sodium hydroxide solution on the viscosity of the paste is obvious. Moreover, from the viscosity contour plot in Figure 3, it can be seen that the intensity of the contour on the vertical coordinate is greater than the intensity of the contour on the horizontal coordinate, indicating that the effect of the ratio of GGBS to PG on viscosity is greater than the effect of the amount of sodium hydroxide solution. Therefore, the maximum value of the viscosity corresponds to the highest point of the response surface, indicating that when the amount of the sodium hydroxide solution is 3.5% and the ratio of GGBS to PG is 1.88, the viscosity of the paste can be optimized to reach the maximum value, and the desirability of the solution is 1.

### 2.4. Fluidity of Composite Material

**Figure 4:**
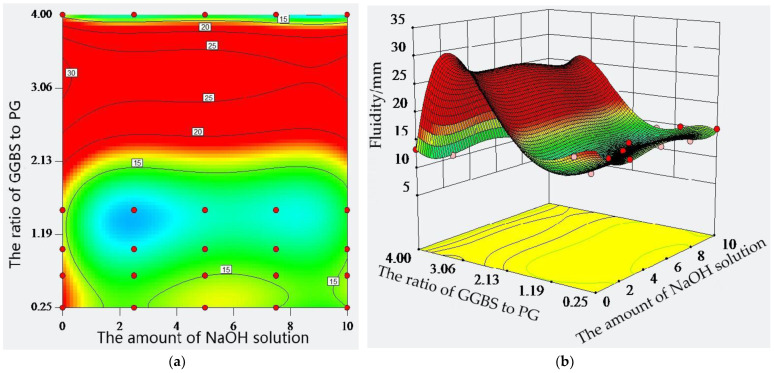
Fluidity of composite material: (**a**) isohypse contour; (**b**) three-dimensional surface.

According to its different roles, the water in the paste is distinguished as bound water and free water. Bound water is also known as chemically bound water, which is consumed in the reaction with the cementitious material. Free water can be divided into filling water and residual water, according to its role in the paste. Filling water is the water that can fill the solid particles. Residual water is the water that can form a film that wraps around the surface of the solid particles, providing fluidity for the cementitious material particles, and its volume directly affects the fluidity of the paste. Therefore, the more residual water, the greater the fluidity of the paste. Filled water is related to the particle type and morphology of the cementitious material, while the residual water is related to the particle shape and specific surface area of the cementitious material. Therefore, the fluidity of the paste is affected by the type of raw material, the amount of water used, and the size and morphology of the particles. The effect of the ratio of GGBS to PG and the amount of sodium hydroxide solution on the fluidity of the paste was discussed, and the results are shown in Figure 4.

Comparing cement, GGBS, and PG, the fluidity of PG is 18.61 mm, which is the largest, and the fluidity of the GGBS is the smallest at 9.95 mm, indicating that there are obvious differences between the fluidities of the PG, GGBS, and cement. When the amount of sodium hydroxide solution was kept constant, the fluidity of the paste first increased and then decreased with the change in the ratio of GGBS to PG. When the ratio of GGBS to PG was 4:1, the fluidity of the paste was 13.35 mm. When the ratio of GGBS to PG was in the range of 4:1 to 3:2, the fluidity of the paste exhibited a rising trend, indicating that the composite of GGBS and PG has a certain effect on the fluidity of the paste, and that there is an optimum amount of GGBS and PG that can cause the paste fluidity to reach a maximum. In a range of a 1:1 to 1:4 ratio of GGBS to PG, the fluidity of the paste showed a small fluctuation and a decreasing trend with an increase in the amount of the sodium hydroxide solution. In general, the effect of the sodium hydroxide solution on the fluidity of the paste could not provide an optimal amount within the test range. The density of the contour line on the ordinate in Figure 4 is greater than the value of its abscissa, which indicates that the ratio of GGBS to PG has a greater effect on the paste fluidity than the amount of sodium hydroxide solution. Although the response of the factors to paste fluidity is convex upward, the ratio of GGBS to PG reaches a certain maximum at 0% of sodium hydroxide solution. Overall, adding the sodium hydroxide solution is not conducive to the development of paste fluidity.

### 2.5. Water Release Ratio of Composite Material

**Figure 5:**
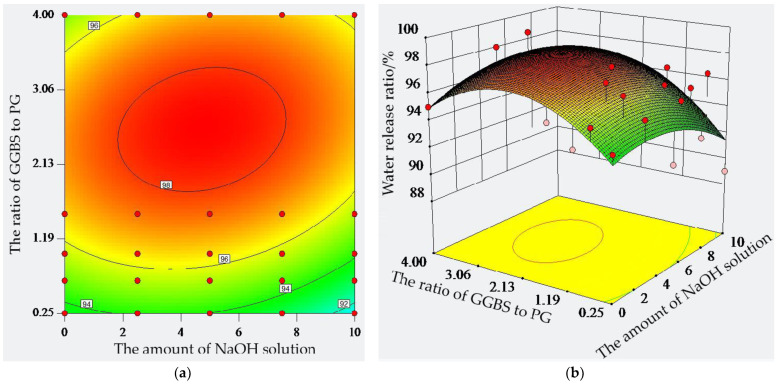
Water release ratio of composite material: (**a**) isohypse contour; (**b**) three-dimensional surface.

The water release ratio of the paste had a direct influence on its performance. For example, in a grouting project, paste with a small water release ratio usually has the advantages of good stability, good dispersion, a dense structure of suspension and solidification body, and small deformation to be able to close the crack well and improve the construction quality of the whole grouting project. Therefore, it is important to study the water release ratio of the paste. The water release ratio of the paste, with different proportions of the composite paste and different amounts of the sodium hydroxide solution, are shown in Figure 5.

The maximum water release ratio in the PG, GGBS, and cement is found for GGBS at 98.6%, and the minimum value is found for PG at 89.7%. This shows that PG, GGBS, and cement have obvious differences in their effects on the water release ratio. When the amount of sodium hydroxide solution was certain, the water release ratio of the paste increased and then decreased with the change in the ratio of GGBS to PG. When the ratio of GGBS to PG was 4:1 and 3:2 and the amount of sodium hydroxide solution was 5%, the water release ratio of the paste reached the maximum at 98.0% and 97.8%, respectively. When the ratio of GGBS to PG was 1:1, 2:3, and 1:4 and the amount of sodium hydroxide solution was 2.5%, the maximum water release ratio of the paste was 97.7%, 97.0%, and 95.6%, respectively. Overall, the higher the content of GGBS, the greater the water release ratio of the paste. The higher the content of PG, the smaller the water release ratio of the paste. When the ratio of GGBS to PG was certain, the water release ratio of the paste increased and then decreased with the increase in the sodium hydroxide solution. For example, when the amount of sodium hydroxide solution was 2.5%, 5%, 7.5%, and 10%, the GGBS to PG ratio reached a minimum at 1:4, which is 95.6%, 91.5%, 92.5%, and 89.0%, respectively. The contour line on the horizontal coordinate is more dense than those on the vertical coordinate; this also indicates that the effect of the amount of sodium hydroxide solution on the water release ratio of the paste is greater than that of the ratio of GGBS to PG. The response of each factor to the paste’s water release ratio is gradually increased, and the highest point of the response surface corresponded to the maximum value of the paste’s water release ratio when the amount of sodium hydroxide solution was 0.0% and the ratio of GGBS to PG was 3.05. The desirability of the scheme was then 0.86.

### 2.6. Water Film Thickness of Composite Material and Influencing Factor Analysis

The water film thickness is the ratio of the remaining water in the paste to the total surface area of the solid particle. It is related to the filling density and the total surface area of solid particle. Under the same water consumption, the greater the filling density, the smaller the water used to fill the particle gap, the more water remaining, and the greater the water film thickness. Under the same residual moisture, the larger the specific surface area, the smaller the average residual moisture per unit area, and the smaller the water film thickness. In order to analyze the effect of the ratio of GGBS to PG on the water film thickness under NaOH excitation, the density of the sodium hydroxide solution used in the test was consistent with that of the water. At the same time, the maximum mass values under different water–binder ratio were tested and calculated to obtain the water film thickness of the paste under different materials and different ratios. The results are shown in Table 1.

As shown in Table 1, among the three materials, PG had the largest water film thickness, followed by GGBS, with cement having the smallest water film thickness. The water film thickness of PG was 3.2 times than that of cement; however, the water film thickness of GGBS was basically the same as that of cement, and the difference was less than 2.1%. Therefore, PG demonstrated the greatest contribution to the water film thickness of the paste. With the addition of PG and mineral GGBS, the water film thickness of the paste increased from 0.481 μm to 1.112 μm, and the maximum increase was nearly three times. Generally speaking, the greater the content of PG, the greater the water film thickness of the paste. In addition, it is not difficult to determine from the calculation in Formula (4) that the water film thickness is affected by the density of the raw materials, specific surface area of raw materials, void volume of raw materials, water consumption, etc., as mentioned later.

Among three materials, the maximum density of cement was 3.073 g/cm^3^, followed by GGBS at 2.893 g/cm^3^ and PG, which had the minimum value of 2.212 g/cm^3^. Due to the volume of material and the density and particle size of the material itself, the same volume of the container was used to test the maximum mass of each material. However, the maximum mass values of the three materials were different; namely, the maximum mass value of cement was 206.92 g, and the minimum mass value of PG was 171.33 g. In addition, the difference of the three material particles also affected the inter-particle volume of each material paste. The minimum inter-particle void volume of the PG was 0.0343 mL, followed by 0.043 mL for cement and 0.0477 mL for GGBS. The larger the volume of inter-particle voids, the larger the volume of filling water required. Therefore, the optimal volume of the water–binder ratio for each material is different: PG has the largest volume of water–binder ratio, following by cement, and GGBS is the smallest.

For the water film thickness of material, in addition to the influence of the remaining water, the total surface area of the composite material is also a concern. For GGBS, PG, and cement, the order of the specific surface area is as follows: PG < GGBS < cement. Therefore, the order of the water film thickness is as follows: PG > GGBS > cement. When GGBS was compounded with PG, the mass of the five test samples upon reaching the most suitable dense state gradually decreased as the amount of PG increased and the amount of GGBS decreased. This is because the density of PG is less than GGBS, leading to the maximum mass value of the material gradually decreasing with the increase in PG. In addition, the specific surface area of GGBS is greater than that of PG; the total surface area of the composite material decreases gradually from 4:1 to 1:4 with respect to the ratio of GGBS to PG, and the water requirement of the material should also be gradually reduced. However, in the range of five test ratios, the actual amount of the trend of first increased and then decreased. This phenomenon is because the filling density of the five test materials showed a trend of first decreasing and then increasing when the ratio of GGBS to PG 1:1 reached a minimum value of 0.580 g/mL, indicating that the volume fraction of GGBS and PG and the change in material density have a greater impact on the filling density of the material. Therefore, the interparticle void volume of the material showed a trend of first increasing and then decreasing, again reaching a maximum value of 0.0417 g/mL at a ratio of GGBS to PG of 1:1. The specific surface area of the material gradually decreased with the increase in PG, indicating that the actual amount of GGBS and PG and the total specific surface area of material affect the formation of voids in material, the void volume, and the thickness of the water film of the material. Therefore, the greater the PG dosage, the greater the water film thickness of the paste, with a maximum of 1.122 μm, which is 2.31 times the minimum water film thickness of the composite material.

**Figure 6:**
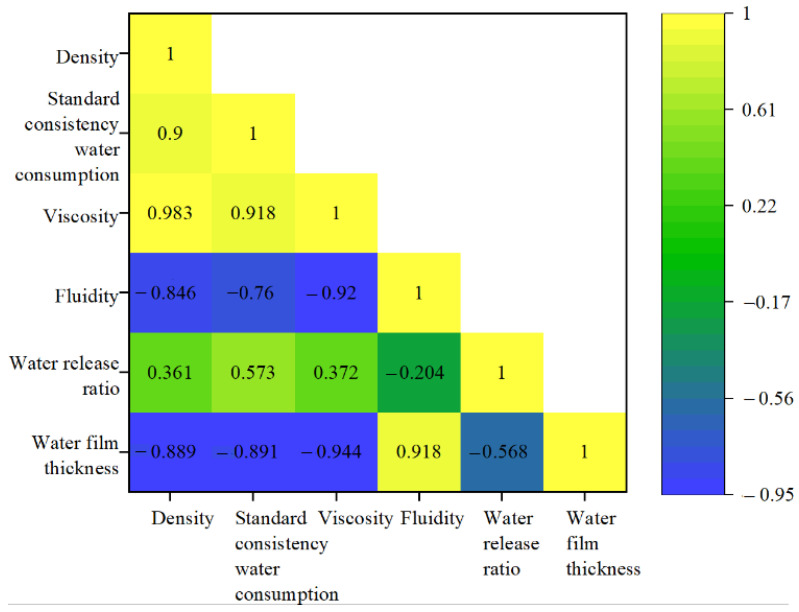
Correlation analysis.

In order to analyze the relationship between the water film thickness and the standard consistency water consumption, viscosity, fluidity, and water release ratio, the results are shown in Figure 6.

As shown in Figure 6, it can be seen that the water film thickness of the paste is negatively correlated with the water consumption of standard consistency, viscosity, and water release ratio, and positively correlated with the fluidity. In addition, can also be found from the above test results that the greater the PG mixture, the greater the water film thickness of the paste. Therefore, in theory, there is an optimal PG content for maximizing the water film thickness, meaning that the free water content attached to the surface of solid particles is the highest at this content; that is, the fluidity of mortar and concrete is the best.

## 3. Materials and Methods

### 3.1. Experimental Materials

**Figure 7:**
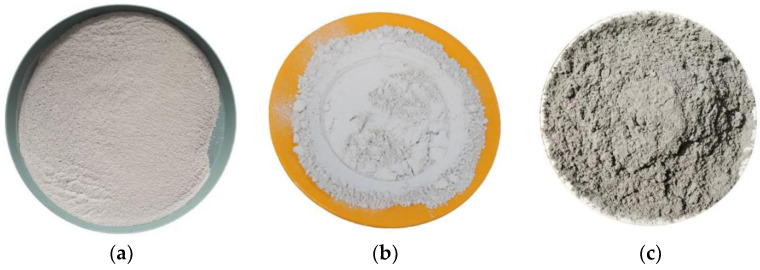
Sample of PC, PG, and GGBS. (**a**) PG; (**b**) GGBS; (**c**) PC.

The PG used in this experiment was obtained from China’s Kailin Chemical Fertilizer Co., Ltd. (Guiyang, China). It had a grey-white appearance, a density of 2.212 g/cm^3^, a specific surface area of 115 m^2^/kg, an initial setting time of 35.40 min, and a final setting time of 42.60 min. Its entire performance index met the Chinese national standard GB/T23456-2018 [28] for second-class. In addition, after ultrasonic water washing, the PG did not contain impurities such as P_2_O_5_, F^−^, Pb, Cr, Ni, or other heavy metals. The GGBS was S105-grade with a density of 2.893 g/cm^3^ and a specific surface area of 460 m^2^/kg. The ordinary Portland cement (PC) was made of P.O. 42.5R, with a density of 3.073 g/cm^3^ and a specific surface area of 380 m^2^/kg. Samples of PG, GGBS, and cement are shown in Figure 7, and their chemical compositions are shown in Table 2. The sodium hydroxide solution used in the experiment was a colorless and transparent liquid with a density of 1 g/cm^3^. The mixing water was laboratory tap water.

### 3.2. Experimental Design

**Figure 8:**
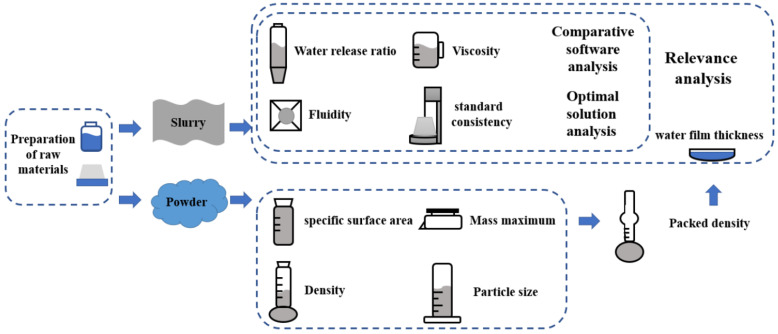
Preparation and characterization of the paste.

The experiment was designed according to different ratios of GGBS to PG (4:1, 3:2, 1:1, 2:3, and 1:4) and different amounts of the sodium hydroxide solution (0%, 1, 2.5%, 5%, 7.5%, and 10%), as shown in Table 3. The preparation process for investigating the effects of the ratio of GGBS to PG and the amount of sodium hydroxide solution on the rheological properties of the paste, such as the water consumption of standard consistency, viscosity, fluidity, water precipitation rate, and water film thickness, is shown in Figure 8.

### 3.3. Test Methodology

#### 3.3.1. Density

The density of three kinds of raw materials and composite materials with different ratios of GGBS to PG was tested according to the GB/T 208-2014 [29] “Test Method for Determination of Cement Density” and calculated according to Formula (1). The density experiment was repeated three times, and the average value was taken as the test result.

(1) ρ=m/V

where *ρ* is the density, g/cm^3^; *m* is the mass of material, g; *V* is the volume of t material.

#### 3.3.2. Standard Consistency Water Consumption

The standard consistency water consumption of three kinds of raw materials and composite materials with different ratios of GGBS to PG was tested according to the GB/T1346-2011 [30] “Cement standard consistency water consumption, setting time and stability test method”. Each experiment was repeated three times, and the average value was taken as the test result.

#### 3.3.3. Viscosity

The viscosity of the paste was measured using a digital display rotational viscometer (Lichen NDJ-9S). After repeated debugging before the test, the No. 2 rotor was used. The selected parameter was 6 r/min, and the full scale was 5000 mPa·s. The specific operation was as follows: before the test, the viscometer used was assembled and adjusted to ensure its normal state. The uniformly stirred paste was then introduced into a beaker with a diameter of 80 mm, which was placed into the No. 2 rotor. The viscometer was then started. The viscosity value was read after the paste was stable or the viscometer had worked for 20 r. Each experiment was repeated three times, and the average value was taken as the test result.

#### 3.3.4. Fluidity

The fluidity of the paste was tested using a frustoconical die with top and bottom diameters of 36 mm and 60 mm, respectively, and a height of 60 mm. The specific test operation was as follows: first, the paste was poured into a frustoconical die that was placed on a flat glass plate, which was then pounded 5 times with a 1 cm wide ruler and smoothed out. Then, the frustoconical die was lifted in the vertical direction. Finally, the maximum diameter in the two perpendicular directions was read with a vernier caliper after 30 s of the paste flowed. Each experiment was repeated three times, and the average value was taken as the test result.

#### 3.3.5. Water Release ratio

An amount of 100 mL of the paste with homogeneous mixing was loaded into a measuring cylinder with a scale of 100 mL. It was ensured that the horizontal liquid level of the paste coincided with the 100 mL scale of the measuring cylinder. After the paste was left to stand for 2 h, the paste stratification scale value (*V*) was read, and the paste’s water release ratio (*η* = 100 − *V*) was calculated. Each experiment was repeated three times, and the average value was taken as the test result.

#### 3.3.6. Calculation of Filling Density and Water Film Thickness

The water film thickness was based on the calculation of the filling density, and the filling density was based on the maximum mass value under different water–cement ratios (*M_max_*). The specific operation was as follows: the water–cement ratio was fixed at 0.2 before the beginning of the experiment, and was gradually increased by increasing the mass of water consumption and fixing the mass of the cementitious material. With the increase in the water–binder ratio, the solid particle concentration of the composite system first increased and then decreased. The peak value was the filling density. The filling density of the three kinds of raw materials and the composite materials was calculated according to Formula (2). Each experiment was repeated three times, and the average value was taken as the test result.

(2) $$P_{max}=\frac{V_{b}}{V}$$

where *P_max_* is the filling density, g/mL; *V_b_* is the volume of the gelling material, mL; *V* is the volume of the test vessel, mL. The volume of the vessel used for the test was 103.378 mL.

In Equation (2), *V_b_* can be calculated using the maximum mass value (*M_max_*). The specific calculation formula is shown in Equation (3).

(3) $$V_{b}=\frac{M_{max}}{R_{m}\rho_{m}+R_{p}\rho_{p}+R_{c}\rho_{c}+V_{w}\rho_{w}}$$

where *V_w_* is the volumetric of the water–binder ratio; *M_max_* is the maximum mass value, g; *R_m_*, *R_p_*, *R_c_,* and 𝜌*_w_* are the volume fractions of GGBS, PG, cement, and water, respectively; 𝜌*_m_*, 𝜌*_p_*, and 𝜌*_c_*, are the density of GGBS, PG, and cement in g/cm^3^, respectively

The water film thickness of the paste can be calculated by the filling density, and the specific calculation process is shown in Equations (4)–(7).

(4) $$T=\frac{w_{e}}{A}$$

(5) $$w_{e}=V_{e}-V_{P}$$

(6) $$v_{p}=\left(\frac{1}{p_{max}}-1\right)\left(\frac{M_{m}}{\rho_{m}}+\frac{M_{p}}{\rho_{p}}+\frac{Mc}{\rho_{c}}\right)$$

(7) $$A=M_{m}S_{m}+M_{p}S_{p}+M_{c}S_{c}$$

where *T* is the water film thickness, μm; *w_e_* is the volume of the remaining water, mL; *V_e_* is the actual volume of water used, mL; *V_p_* is the volume of the inter-particle voids, mL; A is the total surface area of the solid particle, m^2^; *M_m_*, *M_p_*, and *M_c_* were the actual mass of GGBS, PG, and cement in g, respectively; *S_m_*, *S_p_*, and *S_c_* were the specific surface area of GGBS, PG, and cement in m^2^/kg, respectively.

## 4. Conclusions

In this work, the effect of PG and GGBS on the standard consistency water consumption, viscosity, fluidity, water precipitation rate, and water film thickness of the cement PG-GGBS system is discussed. The main experimental results can be drawn as follows:

(1) The density of the mixture with GGBS and PG gradually decreases with the decrease in GGBS content and the increase in PG content. Compared with the density at 4:1, the density of the mixture at the ratios of 3:2, 1:1, 2:3, and 1:4 decreases by 2.70%, 10.64%, 13.37%, and 15.86%, respectively. The standard consistency water consumption of the paste shows ups and downs after adding a sodium hydroxide solution, GGBS, and PG, and they have similar influence laws on the standard consistency water consumption of the paste;
(2) The viscosity of the paste with GGBS and PG at ratios of 4:1, 3:2, 1:1, 2:3, and 1:4 gradually decreases by 15.5%, 32.1%, 36.1%, and 46.8%, respectively, compared with the GGBS and PG ratio of 4:1. The ratio of GGBS to PG has a greater effect on the viscosity than the amount of sodium hydroxide solution;
(3) The compounds of PG and GGBS and the amount of sodium hydroxide solution have a significant effect on the fluidity of the paste, and the ratio of GGBS to PG has a greater effect on the fluidity of the paste than the amount of sodium hydroxide solution. Therefore, the larger the amount of sodium hydroxide solution is, the more unfavorable conditions are to the fluidity and viscosity of the paste;
(4) The effect of the amount of sodium hydroxide solution on the water release ratio of the paste is greater than that of the ratio of GGBS to PG. Overall, the higher the content of GGBS, the greater the water release ratio of the composite paste, and the higher the content of PG, the smaller the water release ratio of the composite paste;
(5) The variation in the ratio of GGBS to PG has a significant effect on the water film thickness of the paste, showing that the greater the PG mixture, the greater the water film thickness of the paste, reaching 1.122 μm, which is 2.31 times the minimum water film thickness of the composite material. At the same time, the water film thickness of the paste is negatively correlated with the standard consistency water consumption, viscosity, and water release ratio, and positively correlated with the fluidity.

Generally, mortar can be regarded as a solid–liquid two-phase system of paste and sand, and concrete is regarded as a two-phase composition of mortar and coarse aggregate. Therefore, the rheology of the paste affects the rheological properties of mortar and concrete. In this experiment, the effects of the ratio of GGBS to PG and the amount of sodium hydroxide solution on the rheological properties of the paste were investigated. It was found that both parameters can affect the water film thickness of the paste. On the whole, the greater the PG mixture, the greater the water film thickness of the paste. Therefore, in theory, there is an optimal PG content maximize the water film thickness, meaning that the free water content attached to the surface of solid particles is the highest at this content; that is, the fluidity of the mortar and concrete is the best. The research results have important reference significance for promoting the development of rheological film thickness theory and the practical engineering applications of PG, which has a positive role in promoting the realization of the Chinese double-carbon strategic task.

## Figures and Tables

**Table 1:** Water film thickness of different materials.

Number	$M_{max}$	*V_w_*	$P_{max}$	$V_{P}$	$w_{e}$	A	T
GGBS	185.96	0.45	0.555	0.0477	32.038	71.703	0.447
PG	171.33	0.30	0.660	0.0343	23.822	16.950	1.405
PC	206.92	0.40	0.576	0.0430	29.494	67.406	0.438
GGBS:PG = 4:1	186.46	0.40	0.609	0.0380	29.499	61.357	0.481
GGBS:PG = 3:2	186.04	0.50	0.603	0.0395	34.420	48.809	0.709
GGBS:PG = 1:1	178.98	0.55	0.580	0.0417	36.641	40.911	0.787
GGBS:PG = 2:3	177.66	0.40	0.618	0.0379	29.499	37.475	0.896
GGBS:PG = 1:4	173.66	0.40	0.624	0.0376	29.499	26.519	1.112

**Table 2:** Chemical composition of PC, PG, and GGBS.

Name	Al_2_O_3_	SiO_2_	Fe_2_O_3_	CaO	MgO	Na_2_O	K_2_O	SO_3_	TiO_2_
PG/wt.%	1.52	8.64	0.12	38.92	0.32	0.22	0.02	50.24	/
GGBS/wt.%	15.75	32.9	0.3	39.78	8.86	0.29	0.43	0.37	1.32
PC/wt.%	7.67	26.1	4.69	54.87	2.91	0.46	0.34	2.96	/

**Table 3:** Experimental scheme design.

Number	GGBS/g	PG/g	Water/g	Sodium Hydroxide Solution/g	Sodium Hydroxide Solution Ratio/%
1	800	200	450	-	0
2	600	400	450	-	
3	500	500	450	-	
4	400	600	450	-	
5	200	800	450	-	
6	800	200	438.75	11.25	2.5
7	600	400	438.75	11.25	
8	500	500	438.75	11.25	
9	400	600	438.75	11.25	
10	200	800	438.75	11.25	
11	800	200	427.5	22.5	5
12	600	400	427.5	22.5	
13	500	500	427.5	22.5	
14	400	600	427.5	22.5	
15	200	800	427.5	22.5	
16	800	200	416.25	33.75	7.5
17	600	400	416.25	33.75	
18	500	500	416.25	33.75	
19	400	600	416.25	33.75	
20	200	800	416.25	33.75	
21	800	200	405	45	10
22	600	400	405	45	
23	500	500	405	45	
24	400	600	405	45	
25	200	800	405	45	

## Data Availability

Data can be obtained from the corresponding authors upon reasonable request.
